# *Implementation Science* and *Implementation Science Communications*: our aims, scope, and reporting expectations

**DOI:** 10.1186/s13012-019-0922-2

**Published:** 2019-08-06

**Authors:** Anne E. Sales, Paul M. Wilson, Michel Wensing, Gregory A. Aarons, Rebecca Armstrong, Signe Flottorp, Alison M. Hutchinson, Justin Presseau, Anne Rogers, Nick Sevdalis, Janet Squires, Sharon Straus, Bryan J. Weiner

**Affiliations:** 1 Department of Veterans Affairs Center for Clinical Management Research, VA Ann Arbor Healthcare System, 300 N. Ingalls Street, Suite 1161, Ann Arbor, MI 48109-5430 USA; 2 Department of Learning Health Sciences, University of Michigan, Ann Arbor, USA; 3 Alliance Manchester Business School, University of Manchester, Manchester, UK; 4 University of Heidelberg, Heidelberg, Germany; 5 University of California, San Diego, USA; 6 Australian Institute of Family Studies, Melbourne, Australia; 7 Norwegian Knowledge Centre for the Health Services, Oslo, Norway; 8 Deakin University, Monash Health, Clayton, Australia; 9 Ottawa Hospital Research Institute, Ottawa, Canada; 10 University of Southampton, Southampton, UK; 11 King’s College London, London, UK; 12 University of Ottawa, Ottawa, Canada; 13 University of Toronto, Toronto, Canada; 14 University of Washington, Seattle, USA

## Abstract

In the 13 years since the inception of *Implementation Science*, we have witnessed a continued rise in the number of submissions, reflecting the growing global interest in methods to enhance the uptake of research findings into healthcare practice and policy. We now receive over 800 submissions annually, and there is a large gap between what is submitted and what gets published. To better serve the needs of the research community, we announce our plans to introduce a new journal, *Implementation Science Communications*, which we believe will support publication of types of research reports currently not often published in *Implementation Science*. In this editorial, we state both journals’ scope and current boundaries and set out our expectations for the scientific reporting, quality, and transparency of the manuscripts we receive.

## Background

**Fig. 1:**
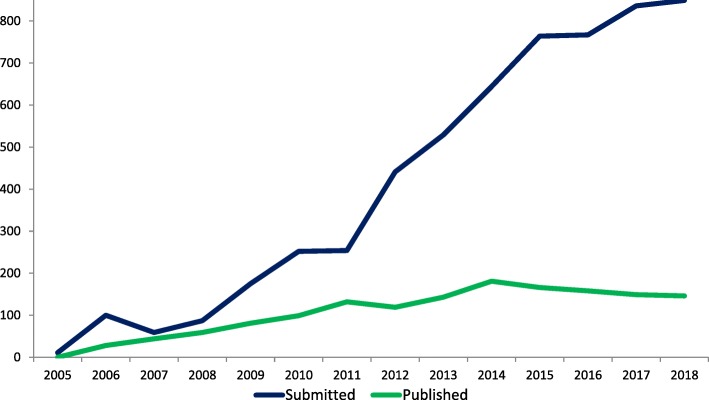
Manuscripts submitted to and accepted for publication in *Implementation Science* 2005–2018

In the 13 years since the inception of *Implementation Science*, we have witnessed a continued rise in the number of manuscripts submitted. We now receive over 800 submissions annually (see Fig. 1), reflecting the growing interest from researchers, funders, and health professionals and policy makers in promoting the uptake of research findings into healthcare practice and policy. The number of manuscripts published in *Implementation Science* has remained rather stable, between 120 and 150 per year.

The large gap between what is submitted and what gets published is driven by two key issues, namely scope and scientific quality. This editorial aims to address both of these issues and act as a further guide to researchers seeking to publish their work in *Implementation Science* and our new companion journal *Implementation Science Communications*.

## Scope and boundaries of *Implementation Science*

In 2017, we reviewed and provided a detailed explanation and elaboration of our journal scope [1]. At that point, we did not expand the boundaries of our scope, and we continue to maintain the same scope. Our focus remains on the publication of studies examining the implementation of evidence-based healthcare interventions, practices, or policies, or the de-implementation of those demonstrated to be of low or no clinical benefit or even harmful. We retain a strong emphasis on reports of studies with strong study design and a high degree of rigor, across both quantitative and qualitative methods, including mixed methods.

For implementation effectiveness, we seek to publish studies that employ rigorous experimental or quasi-experimental designs regardless of whether they report effects or no effects. By rigorous, we mean those designs that would be eligible for inclusion in Cochrane EPOC reviews [2]. This can include type 2 or type 3 hybrid designs where there is a dual a priori focus on assessing clinical effectiveness and implementation strategies [3], but only where there is a clear justification and major component of implementation research. Type 2 hybrid designs have a dual focus on effectiveness and implementation outcomes, for example, testing both the effectiveness of brief cognitive behavioral therapy and the implementation strategies [4]. Type 3 hybrid designs have a primary emphasis on evaluating implementation, for example of a diabetes prevention program [5]; in such studies, data on clinical outcomes are also collected, as secondary or tertiary endpoints.

In addition to the above, we continue to receive a considerable number of manuscripts reporting studies testing novel clinical, service, or population health interventions; in such studies, the effectiveness of the intervention or practice has yet to be established. As our scope focuses on the implementation of interventions of demonstrated effectiveness, we routinely reject these manuscripts, offering transfer to other BMC journals. These exclusion criteria also apply to type 1 hybrid designs where the focus is on testing the effects of a clinical intervention on relevant outcomes while observing and gathering descriptive information on implementation [3]. Studies of this type fall outside of our journals’ scope.

Implementation interventions are invariably complex, and so, alongside a rigorous evaluation of implementation effectiveness, we also welcome economic evaluations [6], process evaluations, and other qualitative research that examine different aspects of how an intervention functions in a given context and which contribute to our overall understanding of effectiveness. This includes the study of adaptation and fidelity, mechanisms of impact, and contextual influences on implementation and outcomes, sustainability, and scalability as well as the study of influences on the provider, patient, and organizational behavior. Crucially, we expect the methods employed in such studies to be an appropriate fit to the question(s) being addressed and to be informed by relevant conceptual frameworks [7–9].

We also welcome articles that present new methods and articles that question or challenge existing implementation policies, practices, evidence, or theory and suggest modifications or alternatives. However, it is worth noting that there is no shortage of frameworks and theories already developed and applied in implementation research [7–9]. So, rather than developing yet more frameworks or theories, our preference is for empirical studies that test and advance our understanding of how best to deploy the existing theoretical base [10]. With debate papers, we reject those that fail to ground the central argument within the existing implementation research literature. Most debate papers are of greater relevance if the arguments posed are based upon systematic reviews of the relevant evidence.

## Aims and scope: *Implementation Science Communications*

We recognize that our current focus in *Implementation Science* on innovative, rigorous, and high-quality papers that contribute significantly and substantially to the accumulated knowledge in the science of implementation, coupled with our current scope, results in frequent rejection and offers to transfer to journals outside the discipline of implementation science. We are aware that many authors would prefer to publish in a journal that specializes in health-related implementation research. We are therefore launching *Implementation Science Communications* in 2019, to accommodate a wider range of types of study reports, and a somewhat broader scope. In doing so, we are emulating the broad goal of the BMC Series in their subject-specific journals, to make decisions primarily on the grounds of scientific validity (sound science), rather than broad interest or likely impact. *Implementation Science Communications* will accept manuscripts for which the audience may be narrower and more focused than the wider community of implementation or dissemination researchers. This includes the opportunity to consider manuscripts in areas that border on the general scope for *Implementation Science* or that are more descriptive than hypothesis-driven.

The new journal will be closely aligned with *Implementation Science*. There will be joint coordination and governance of both titles, and the co-Editors-in-Chief of *Implementation Science Communications* will be closely aligned with those of *Implementation Science*. All bring a commitment to the continued growth and development of the field of implementation research.

To clarify the scope and focus of the two related journals, Table 1 presents the types of manuscripts likely to be accepted by or rejected from *Implementation Science* and discusses how some manuscripts might fit well within *Implementation Science Communications*. This should assist prospective authors to judge which journal is the most suitable home for their implementation research.

**Table 1:** Factors promoting the likelihood of acceptance or rejection from *Implementation Science* and *Implementation Science Communications* by manuscript type

Type of manuscript	Factors promoting the likelihood of acceptance in *Implementation Science*	Factors promoting the likelihood of rejection from *Implementation Science*	Required reporting guideline checklist	Possibility of acceptance in *Implementation Science Communications*
Effectiveness	Studies that fit our journal scope and that employ rigorous experimental or quasi-experimental designs (i.e., designs eligible for inclusion in Cochrane EPOC reviews) and evaluate the implementation of an evidence-based practice or policy, or de-implementation of those demonstrated to be of low or no clinical benefit	Studies which lack a rigorous experimental study design such as quality improvement reports, service evaluations, or uncontrolled before-after studies	CONSORT for trials	Observational outcome studies, for example, those that use case study design, smaller pilot studies, pre-implementation studies, studies focused on dissemination using innovative approaches, and/or descriptive studies
		Studies evaluating the effectiveness of novel clinical, organizational, public health, or policy interventions		
Economic evaluation	Any cost-effectiveness analysis that compares the costs and outcomes of two or more implementation strategies	Cost and cost consequences’ analysis where disaggregated costs and outcomes are presented	CHEERS	Costing analyses that do not provide clear effectiveness findings but exemplify methods for economic study in implementation and dissemination; descriptive cost analyses
Implementation intervention development reports	Prepared and submitted prior to the reporting of the effectiveness of the intervention	Post hoc submission (submitted after the reporting of the effectiveness of the intervention)	TIDIER, STARI	*Implementation Science Communications* will also consider reports of intervention development after reporting on effectiveness in some cases
	Plans for (robust) evaluation are made explicit	No plans for (robust) evaluation		
	Providing empirical and/or theoretical rationale, typically using a stepwise approach	Non-transparent linkages between interventions and preceding analysis		
Methodology	Articles that present methods which may either be completely new or offer an improvement to an existing method	Descriptive accounts of largely established methods without any associated novel methodological insights	N/A	Descriptive implementation methods, which offer high-quality application of existing models, theories, and frameworks within specific health settings
	Articles reporting empirical comparisons of one or more methodological approaches or which clearly state what they add to the existing literature			
Implementation pilot and feasibility studies	Studies that fit our journal scope and are conducted with the explicit purpose of assessing feasibility and planning for an intervention that is expected to contribute to existing knowledge	No justification for conduct	CONSORT pilot and feasibility study checklist	Well-conducted pilot and feasibility studies providing important pilot outcomes: effect size estimates, contextual factors, assessment of determinants of implementation, feasibility, acceptability, and other implementation-focused outcomes
		Over claim on the basis of results		
	Studies indicating how a subsequent study will draw from the pilot study			
	Clear plans for further evaluation or where there are clear reasons for not			
Implementation process evaluation	Studies that fit our journal scope and are submitted contemporaneously with or following reports of intervention effectiveness and that take account of the main evaluation outcomes	Process evaluations submitted in advance of the conduct of the main effectiveness analysis (it cannot be clear if they are explaining an effect or the absence of an effect)		Implementation process evaluation reports that reflect lessons learned that may generalize to other work
				Process evaluations of complex clinical or preventive interventions, which have substantial implementation challenges (e.g., in the context of clinical trials) may also be published
	Studies evaluating the fidelity of implementation, mechanisms of impact, and or contextual influences on implementation and outcomes	Process evaluations that do not take account of the main evaluation outcomes		
		Process evaluations of clinical or preventive interventions		
Protocols	Protocols for innovative or very large scale studies that fit our journal scope and inclusion criteria for rigorous study designs with an emphasis on experimental design, that have been through a competitive peer review process to receive funding from a nationally or internationally recognized research agency, that have received appropriate ethics review board approval, and that have been submitted within 12 months of ethics approval	Protocols that have not been the subject of peer review by a national or international research agency	SPIRIT	Protocols for pilot and feasibility studies and smaller scale studies that fit our journal scope and inclusion criteria for study designs, including quasi-experimental and other study designs. We may accept protocols for multi-site quality improvement or service evaluations if they meet other criteria below, that have been through a competitive peer review process to receive funding from a regionally, nationally, or internationally recognized research agency, that have received appropriate ethics review board (or equivalent) approval, and that have been submitted within three possible time points: (1) within 3 months of ethics approval, (2) prior to enrolment of the first participant/cluster, and (3) before the end of participant/cluster recruitment (i.e., prior to the commencement of data cleaning or analysis)
		Protocols that have not received ethics review board approval		
		Protocols for quality improvement or service evaluations, which lack a rigorous study design		
		Protocols for pilot or feasibility studies		
		Protocols for systematic reviews and other types of synthesis focused on implementation or dissemination research		
		Protocols that are submitted for studies where data cleaning and analysis have begun		
Qualitative and mixed methods studies	Studies that fit the journal scope and meet applicable criteria for quality and validity	Studies where there are doubts about whether planned data saturation has been achieved	COREQ or RATS	Studies that focus on smaller samples or rely on descriptive qualitative methods only; mixed methods studies with appropriate design
		Single-site case studies with limited typicality or transferability		
		Studies that fail to link to relevant theory or, without contextualization and with little reference to previous relevant qualitative studies or reviews		
Short reports	Brief reports of data from original research which present relatively modest advances in knowledge or methods	Reports of meetings, “doing implementation” or “lessons learned”	N/A	The initial scope of *Implementation Science Communications* may include meeting short reports and brief descriptions of lessons learned
Systematic reviews and other syntheses	Systematic reviews and other types of synthesis (such as rapid, realist, or scoping) that fit our journal scope and which may cover issues such as the effects of implementation interventions and influences on the uptake of evidence	Non-systematic or narrative literature reviews that fail to use explicit methods to identify, select, and critically appraise relevant research	PRISMA	Narrative and other types of reviews may be accepted
			RAMESES for realist reviews	
		Reviews and syntheses that fail to adhere to recognized quality and reporting standards		
Research on education in implementation science	Empirical evaluation of training programs and materials for *Implementation Science*	Description of educational programs and materials		*Implementation Science Communications* may also publish pilot and small studies in this domain
Debate	Papers which question or challenge existing implementation policies, practices, evidence, or theory and suggest modifications or alternatives; clearly contextualized in the current literature	Papers which fail to contextualize in the literature or demonstrate how they build upon the existing implementation research literature; unlikely to be accepted in *Implementation Science* if not based on a systematic review of the literature	No checklist required; however, note preference for reviews over pure debate	Non-systematic review-based papers may be accepted in *Implementation Science Communications* if interesting concepts are discussed and/or the authors make the case that a systematic review is not feasible or appropriate

### Sound science

Alongside failure to meet scope requirements, poor scientific quality remains a common reason for rejection. Promoting the development, refinement, and quality of implementation research was a key aim of the founding Editors [11] and remains so today [1]. Across both *Implementation Sci*ence and *Implementation Science Communications*, we will support and promote efforts to improve research quality and transparency as components of sound science.

### Prospective trial registration

We support initiatives to improve the reporting of randomized trials. We have adopted the ICMJE recommendation [12] and normally consider for publication trials that have been registered with an appropriate publicly available trial database prior to enrolment of the first participant/cluster. Our expectation is that all trials will be prospectively registered.

While there are no fixed rules about the registration of other study designs, we strongly encourage authors of other outcome evaluations to register studies whenever possible. Researchers undertaking systematic reviews are advised to prospectively register their review with PROSPERO or another publicly accessible registry.

### Enhancing research reporting

Over the last decade, we have routinely required authors submitting manuscripts reporting trials to *Implementation Science* to complete the CONSORT checklist or relevant extension. Similarly, a requirement to complete the PRISMA checklist has been enforced for authors submitting systematic reviews. No other checklists have been routinely or uniformly enforced. As a journal that receives manuscripts covering a wide range of study designs, variation in the standards of reporting of the research that we publish has been the result.

Because our aim is to promote research quality and transparency, as an aid to our readers, reviewers, and Editors, we now require authors submitting manuscripts to both journals (regardless of study design) to complete and include a design appropriate reporting checklist. This is true of both *Implementation Science* and *Implementation Science Communications.*

The website of the EQUATOR Network provides details of available reporting guidelines (www.equator-network.org). Authors of manuscripts (regardless of study design) should refer to EQUATOR and ensure that they complete and include a design appropriate reporting checklist with their submission. Table 1 includes details of our preferred reporting formats; for those research types where consensus is lacking on reporting format (for example, in qualitative research), we encourage authors to select their preferred checklist.

Improving the quality of intervention description is as much an issue for implementation research as it is for other evaluations of complex interventions. Without sufficient detail, it is difficult for readers to determine what was actually implemented and/or for other researchers to use or replicate the intervention in other studies. While TIDieR is most often used in conjunction with the CONSORT guidelines for trials [13], improved intervention description is relevant across all evaluative study designs [14]. Other relevant standards for reporting implementation interventions (Standards for Reporting Implementation studies - StaRI) and for reporting behavior change interventions (Workgroup for Intervention Development and Evaluation Research - WIDER) have been developed and are available. We strongly encourage authors to use the website of the EQUATOR Network to select their preferred guideline to enhance reporting of interventions.

### Contribution to the field

With all submissions, we expect authors to clearly articulate what is already known and what their work adds to existing knowledge, theory, and thinking in the field. Many submissions currently fail to set the work in the context of the existing literature, and so, we will continue to reject manuscripts that do not clearly build on current knowledge and understanding or appear to provide limited contributions.

We are now requiring all submissions to include a brief, bulleted statement (maximum 100 words) that describes what the paper adds to knowledge in the disciplines of implementation or dissemination science. We will use this information, which should not be a re-statement of the abstract, to evaluate priority for review and in assessing whether a manuscript submitted to *Implementation Science* should be transferred to *Implementation Science Communications*. As noted above, both journals require this information.

### Open science

As open access journals (with open peer review), we are committed to making research and the datasets upon which it is based, publicly accessible. A number of different data sharing approaches have now been adopted across the health and medical literature [15]. We have adopted the policies on data availability of our publisher BMC. As part of online article submission, we now ask authors to include an “Availability of data and materials” section in their manuscript detailing the conditions under which the data supporting their findings can be accessed. Authors who do not wish to share their data must include a formal statement that data will not be shared and explain why. Full details of BMC policies can be found under the submission guidelines section of our website. Again, this is true for both journals.

## Conclusion

In this editorial, we have set our expectations for the scientific reporting, quality, and transparency of the manuscripts we currently receive in *Implementation Science,* and we expect to receive in *Implementation Science Communications*. We encourage prospective authors to familiarize themselves with the journal scope and boundaries before making a submission and to consider carefully which one of the two journals offers the best fit for a study’s scope and methods. We look forward to the next decade as the field continues to grow and evolve and to receiving research that continues to enhance the uptake of evidence-based practices or policies to improve the quality and delivery of healthcare.

## Data Availability

Not applicable
